# Complete protein assignment from sets of spectra recorded overnight

**DOI:** 10.1007/s10858-019-00226-8

**Published:** 2019-02-15

**Authors:** Jonas Fredriksson, Wolfgang Bermel, Martin Billeter

**Affiliations:** 1 Department of Chemistry and Molecular Biology, University of Gothenburg, 40530 Gothenburg, Sweden; 2 Bruker BioSpin GmbH, 76287 Rheinstetten, Germany

**Keywords:** Flexible characterization, Fast data acquisition, Projection, Decomposition, NMR assignment

## Abstract

A flexible and scalable approach for protein NMR is introduced that builds on rapid data collection via projection spectroscopy and analysis of the spectral input data via joint decomposition. Input data may originate from various types of spectra, depending on the ultimate goal: these may result from experiments based on triple-resonance pulse sequences, or on TOCSY or NOESY sequences, or mixtures thereof. *Flexible* refers to the free choice of spectra for the joint decompositions depending on the purpose: assignments, structure, dynamics, interactions. *Scalable* means that the approach is open to the addition of similar or different experiments, e.g. larger proteins may require a wider selection of triple-resonance based experiments. Central to the proposed approach is the mutual support among the different spectra during the spectral analysis: for example, sparser triple-resonance spectra may help decomposing (separating) spin systems in a TOCSY or identifying unique NOEs. In the example presented, backbone plus side chain assignments of ubiquitin were obtained from the combination of either two or three of the following projection experiments: a 4D HCCCONH, a 4D HNCACO and a 3D HNCACB. In all cases, TOCSY data (4D HCCCONH) proved crucial not only for the side chain assignments, but also for the sequential assignment. Even when total recording time was reduced to about 10 h, nearly complete assignments were obtained, with very few missing assignments and even fewer differences to a reference.

**Electronic supplementary material:**

The online version of this article (10.1007/s10858-019-00226-8) contains supplementary material, which is available to authorized users.

## Introduction

NMR applied to proteins is a very versatile tool: It allows investigations on 3D-structure and dynamics as well as binding studies (several reviews have been published recently, for example Sugiki et al. 2017, 2018; Ban et al. 2017). Important goals in modern NMR are reductions of costs and effort, e.g. requiring fewer samples, smaller amounts, less instrument time etc., as well as robustness and reliability also in difficult cases such as (partly) unfolded proteins. Many “fast” NMR approaches have been and are still being proposed, often based on non-uniform sampling (NUS), involving random elements when choosing FIDs to be recorded (for overviews see Delaglio et al. 2017; Eghbalnia and Markley 2017; special issue: non-uniform sampling in biomolecular NMR (Billeter 2017). Another approach is to record projection planes with more or less freedom in selecting projection angles (Eghbalnia and Markley 2017; Murrali et al. 2018; Hiller et al. 2008; Malmodin and Billeter 2006). Data monitoring during acquisition may further save time (Isaksson et al. 2013). Normally, a first step in NMR projects involves obtaining resonance assignments, and many methodological projects are demonstrated on backbone assignments, making use of triple-resonance experiments or their extensions into higher dimensions. While many novel methods have been demonstrated mostly for backbone assignments, fewer examples concern the next steps of a NMR project, assignment of side chain resonances with typically some version of a TOCSY, or experiments for structure and/or interactions.

Our approach consists of selecting a series of experiments necessary for a project, for example by combining assignment experiments based on scalar coupling with experiments based on NOE coupling for structure and/or interactions (Fredriksson et al. 2012a). Spectral data from all these experiments is then jointly analyzed via decomposition, where a major demand is that they support each other. Instead of recording a high-dimensional experiment we record a series of 2D projection planes in order to drastically reduce the number of FIDs acquired. [Note that this approach is orthogonal to methods like SOFAST (Schanda and Brutscher 2005), i.e. these approaches can be combined.] Planes from various experiments can then be combined into a single input stack for simultaneous decomposition. Mutual support achieved in this manner can be illustrated by a simple example: two amide groups with strongly overlapping ^15^N-HSQC peaks may have very different side chain environments while backbone couplings may involve very similar resonances (Fredriksson et al. 2012b). The separation of the two spin systems with the help of TOCSY information may thus be more reliable and support the simultaneous separation of triple-resonance type signals. The projection-decomposition approach has been described earlier (Malmodin and Billeter 2005, 2006; Billeter et al. 2007), and here we only repeat the main relation between input (recorded) projection planes P_i_ and output components, which in turn are separated into shapes *F*_*k*_*(ω)*:

$${P_i}({\omega _p},{\omega _d})={\Sigma _k}\{ [{F_k}({\omega _1})*{F_k}({\omega _2}) \ldots ]({\omega _p}) \otimes {F_k}({\omega _d})\} +\varepsilon$$

*P*_*i*_: projection plane *i* (between 13 and 19 planes were used here; in some earlier applications this number exceeded 50); *ω*_*p*_: indirect axis of *P*_*i*_; *ω*_*d*_: direct axis; *k*: running index over components, which are enclosed in {}; *ω*_1_, *ω*_2_: spectral axes; *F*_*k*_*()*: shape of component *k* along a given spectral axis; “*” denotes the folding operator and “⊗” a direct product; *ε* is a residual that typically collects noise from the input data (Malmodin and Billeter 2006). Note that all *P*_*i*_ are 2D and all *F*_*k*_*()* are 1D; *ω*_*d*_ is often but not necessarily the axis of HN. An illustration (which is discussed in detail later) is given in Fig. 4: The eight shapes (eight panels of this figure) are for: HN and N of Thr12, aliphatic carbons (“Cali”) and aliphatic hydrogens (“Hali”) in Lys11, and for both Lys11 and Thr12: Cα and CO from a HNCACO (“CA” and “CO”), and Cα and Cβ from a HNCACB (“CAb” and “CaB”). Important features are that (a) the projection planes *P*_*i*_ represent the complete and only input to the decomposition, (b) the output are *components* that are reminiscent of (generalized) spin systems, and (c) the components are defined by *shapes* (Staykova et al. 2008a), which look like 1D cross sections (but are defined for one “spin system” only due to the decomposition) and provide directly all requested resonances.

The NMR approach proposed here is both *scalable* and *flexible. Scalable* refers to options of including more data when a more complex protein requires this; this can consist of additional, different experiments or simply increasing S/N or resolution within a given experiment. *Flexible* means that different purposes (assignments, structure, dynamics, interactions) can be accommodated by selecting appropriate spectra. The experiments can be run sequentially, as in the case described here, where all experiments were run directly one after the other. Alternatively, they could be run interleaved, or experiments could be added at a later stage (making sure to reproduce sample conditions). Earlier, we have used projection-decomposition for backbone assignments combining four- and five-dimensional experiments (Staykova et al. 2008a; Fredriksson et al. 2012b), side chain assignments (Fredriksson et al. 2016) or for structure characterization (Fredriksson et al. 2012a, 2016). A preliminary example of flexibility was presented by a joint decomposition including projection planes from experiments for several purposes, resulting in 15-dimensional components (Fig. 1 in Fredriksson et al. 2012a), with shapes along axes for individual backbone atoms as well as along TOCSY and NOESY axes. Here, the main objective is to reduce overall recording time by running joint decompositions of projection planes from different experiments. We achieve two goals: less complex experiments (e.g. with fewer signals) help with the decomposition of more complex ones, and the TOCSY-type data are used *both* for side chain assignments as well as for proper sequential connections of the spin systems. Flexibility and scalability of the approach mean several things: (1) we may vary the combination of experiments used (although TOCSY-type information is required for side chain assignments). (2) We may add more experiments (e.g. more triple-resonance derived experiments to resolve ambiguities); in fact, the final results show that an additional experiment with a shorter TOCSY mixing time may have led to more complete assignments of β-CH groups. (3) Investment in experiment time could be adapted when planning a project, e.g. the number of scans for each FID could be increased, in particular if a NOESY-type experiment is included.

**Fig. 1:**
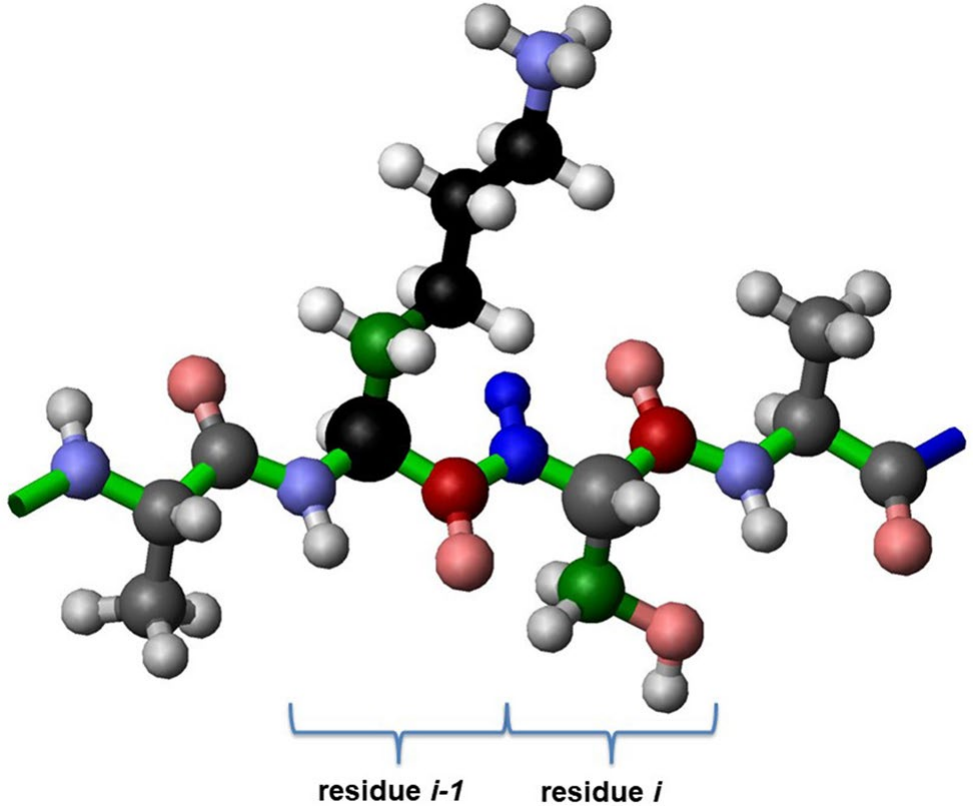
Protein fragment with four residues; of interest are the central ones labelled “*i − 1*” and “*i*”, where residue *i − 1* is a Lys. The backbone runs horizontal and is indicated by green bonds. All experiments have the HN of residue *i* as direct dimension; also always recorded is the corresponding nitrogen (both atoms are represented by dark blue sphere). The experiments record the following nuclei. The 4D TOCSY connects the (blue) amide group of residue *i* with the aliphatic carbons (black, one dark green sphere) and hydrogens (white) of residue *i − 1*. The 4D HNCACO connects the same amide group to the α-carbons (black and grey with increased radius) and carbonyl-carbons (dark red) of both residues *i* and *i − 1*. Similarly, the 3D HNCACB connects the amide group of residue *i* to the same α-carbons and to the β-carbons (dark green spheres of both residues *i* and *i − 1*). (Oxygens are represented by light red spheres.) Figure created with Molmol (Koradi et al. 1996)

We explore here option (1) by three different combinations composed from projections taken from three experiments, and reducing the required total experiment time needed for backbone plus side chains assignments of an easy protein (ubiquitin) to about 10 h. The basis for the time savings is that projections reduce time for a 4D experiment by a factor larger than 1000; depending on which projections are recorded and used, this factor can exceed 2500 (Table S1). More complex proteins (size, disorder …) would likely require variations with respect to options (2) and (3). The applications demonstrated here concern the resonance assignments of both backbone and side chains (the latter includes aliphatic CH_n_ groups, but no aromatic groups and no side chain NH_n_ groups). Because we aim to investigate to what extent the data used (i.e. the experiment combinations) are sufficient, we implemented a series of programs for the current assignment problem. Since these programs serve only one of several possible purposes, namely resonance assignments, (other purposes, for which we propose our approach, may concern dynamics, structures, interactions), they are not central to this presentation of a novel approach. Instead of optimizing them for difficult situations, we rather avoided sophistication and thus could reduce the number of parameters; for example, for a peak picker (in 1D shape) line widths and similar considerations were ignored. (The present study does not use any NOESY data, but this NOESY projections have been used in decompositions and subsequent analysis in earlier publications: Fredriksson et al. 2012a, 2016).

## Methods

Three different input sets were tested in order to achieve complete backbone and side chain assignments of ubiquitin. These input sets in turn were compiled from three different experiments: a 4D HCCCONH (Montelione et al. 1992; Grzesiek et al. 1993; hereafter called TOCSY), a 4D HNCACO (Clubb et al. 1992; Kay et al. 1994) and a 3D HNCACB (Wittekind and Mueller 1993; Muhandiram and Kay 1994). All three experiments were recorded as a series of projection planes. Figure 1 illustrates the three experiments for the inner two residues of a four-residue fragment, where the second residue is numbered *i − 1* and the third *i*. All experiments include the amide group of residue *i* (dark blue spheres), with the HN resonances recorded as direct dimension. The 4D TOCSY connects the aliphatic hydrogens of residue *i − 1* (white) with the aliphatic carbons of the same residue *i − 1* (black, one dark green sphere) and with the amide group of residue *i* (dark blue). The 4D HNCACO includes the same amide group, which is connected to the α-carbons (black and grey with increased radius) and carbonyl-carbons (dark red) of both residues *i* and *i − 1*. Similarly, the 3D HNCACB connects the amide group of residue *i* to the same α-carbons and to the β-carbons (dark green spheres) of both residues *i* and *i − 1*.

In a 4D experiment there are 13 possible planes with angles of 0°, ± 45° or 90° to the frequency axes, and in a 3D experiment there are four (Tables S1, S2). In our case, i.e. with 64 complex points recorded in all indirect dimensions and a phase cycle with 16 scans, recording of each plane required just above 1 h (Table S3; times taken from the experiment log files). Thus, the required experimental (instrument) time for 4D projection experiments is about 13 h, and for 3D projection experiments about 4 h. By de-selecting some projection planes (see below and Table 1), the times for the 4D experiments could be reduced to 6–7 h.

**Table 1:** Projection planes used in three different experiment combinations

Projection planes (indirect dim.)^a^	Experiment combinations^b^		
	OB	B	O
Hali	x	x	x
Cali	x	x	x
N (TOCSY)	x	x	x
N + Hali	x	x	x
N − Hali	x	x	x
N + Cali	x	x	x
N − Cali	x	x	x
CO	x		x
CA	x		x
N + CO	x		x
N − CO	x		x
N + CA	x		x
N − CA	x		x
CAb	x	x	
N + CAb	x	x	
N − CAb	x	x	
CaB	x	x	
N + CaB	x	x	
N − CaB	x	x	

^a^Projection planes are indicated by listing indirect dimensions separated by “+” or “−”. Names of indirect dimensions are: N for ^15^N-axis, CA for alpha-carbon from HNCACO, CO for carbonyl carbon from HNCACO, CAb for alpha-carbon from HNCACB, CaB for beta-carbon from HNCACB (the latter two are separated due to the different signs of the signals), Cali for aliphatic carbons of the TOCSY, and Hali for aliphatic hydrogens of the TOCSY

^b^Combinations are: “OB”: projection planes from TOCSY and HNCACO and HNCACB; “O”: TOCSY and HNCACO, “B”: TOCSY and HNCACB. Only the projection planes marked by “x” were input to the decompositions of a given combination

The three input sets consisted each of selected planes from the three experiments (Table 1). The first set is a combination of planes from all three experiments (combination referred to as “OB”), the second is a combination of planes from all the TOCSY and the HNCACB (combination referred to as “B”), and the third is a combination of planes from all the TOCSY and the HNCACO (combination referred to as “O”). The following considerations were applied to the plane selection, with the overall idea to save time by eliminating redundant or noisy data; but in all cases sets of planes that were recorded as a single hyper-complex data set (Table S2) were either all kept or all eliminated. Because all three experiments include a ^15^N-HSQC plane, only the one from the TOCSY was kept. From both 4D experiments, the planes with evolution on all nuclei were not considered due to their lower S/N, and so were planes with no evolution on the nitrogen (that is common to all experiments). This resulted in the use of 19 planes for the combination “OB”, 10 planes for the combination “B”, and 13 planes for the combination “O” (Table 1).

**Fig. 2:**
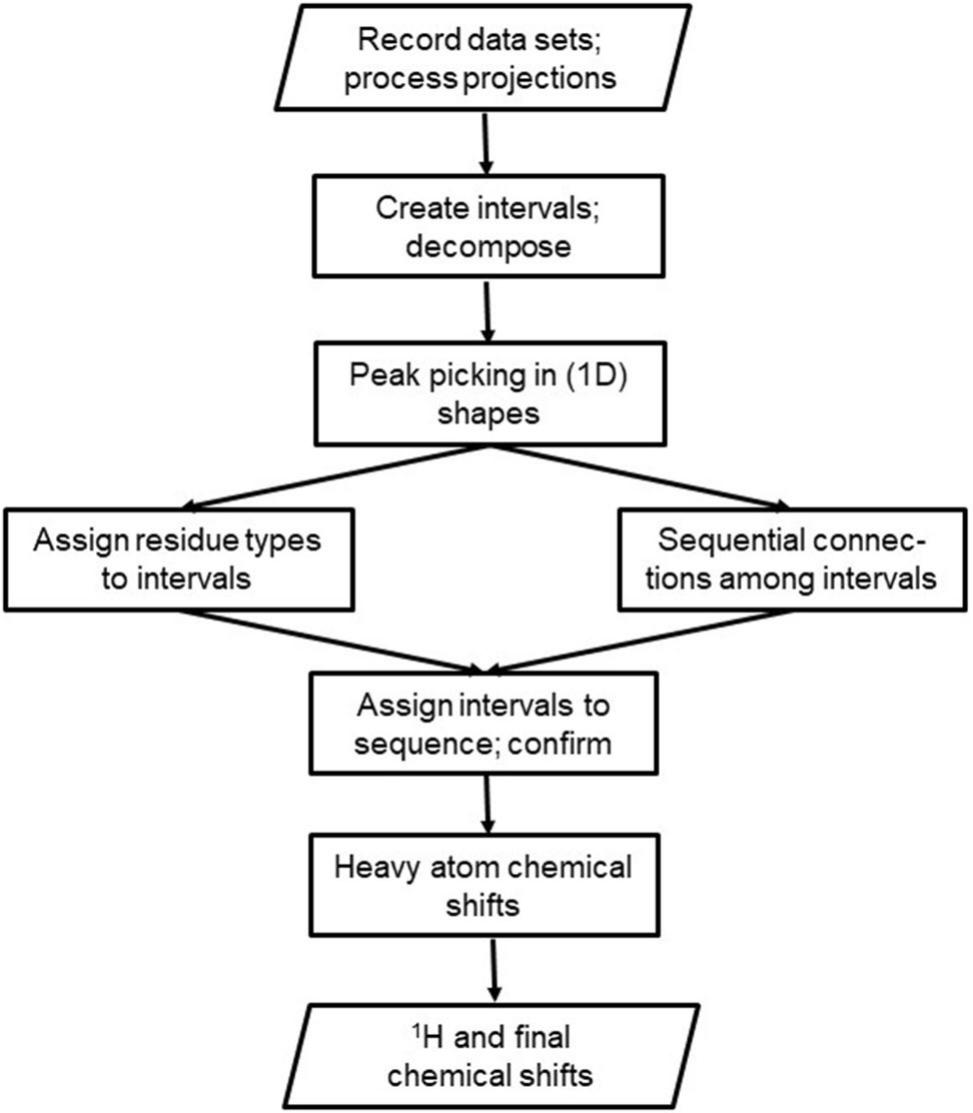
Flow-chart of the assignment process, starting with spectra recording and ending with a final list of chemical shifts for both the backbone and the side chains; see text for explanations

Attempts of complete assignment with each of the three combinations of experiments followed the procedure presented in the flow-chart of Fig. 2: after recording and processing of the experimental data, intervals were defined for all ^15^N-HSQC peaks (using the ^15^N-HSQC plane from the TOCSY) and decomposed, peaks were picked in the resulting (1D) decomposition shapes, possible residue types were identified for each interval and, independently, sequential relations between intervals were determined, intervals were assigned to sequence residues, chemical shifts were determined for all heavy (backbone and side chain) atoms, and finally also for hydrogen atoms. The latter only, i.e. the assignment of hydrogen atoms, in part uses the earlier published DIADECOMP algorithm (Fredriksson et al. 2016). Below, some details are given for each box in Fig. 2.

### Record data sets and process projections

All experiments, which consisted of recording a series of 2D projections, were performed on a 600 MHz AVIII Bruker instrument using an H–C, N TXI room temperature probe, with a doubly labelled 0.5 mM ubiquitin sample (from Silantes). Initially, all projections were recorded: 13 projections per 4D experiment and 4 projections per 3D experiment (Table S1). However, for the decompositions several projections were excluded (see above).

**Fig. 3:**
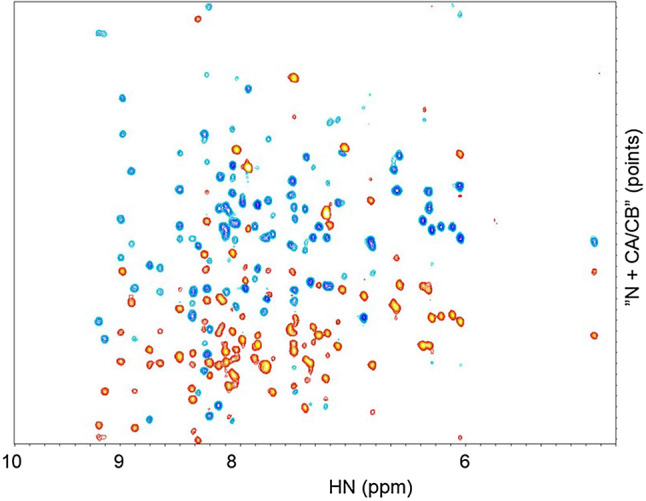
Example input projection plane taken from the HNCACB experiment. The horizontal axis is the direct HN dimension, the vertical axis is a diagonal axis combining the shifts of the nitrogen nuclei and of the Cα and Cβ carbons. Signs of signals are determined by the carbon type: positive for Cα (red) and negative for Cβ (blue). Figure created with nmrDraw (Delaglio et al. 1995)

All recorded data were processed in the same way. Due to the hyper-complex nature of the experimental data, planes involving the same set of nuclei but different signs in the linear combinations were recorded as one dataset (Table S2), which thus needed to be split into individual planes. After this splitting, the same constant phase correction in the direct dimension (derived from inspection of the TOCSY ^15^N-HSQC plane) was applied to all planes, and indirect directions were supplemented with 32 points of linear prediction. After zero filling (yielding 192 points in the indirect dimensions) and Fourier transform the point resolution in ppm was 0.0087 (direct HN), 0.21 (N), 0.43 (C aliphatic), 0.086 (H aliphatic), 0.17 (CA in HNCACO), 0.084 (CO) and 0.42 in carbon of the HNCACB (see Table S4 for spectral widths and offsets). Signals in the HNCACB change sign between Cα and Cβ. However, the decomposition algorithm used considers only non-negative data (Bro and De Jong 1997). Thus, the planes from the HNCACB involving the carbon dimension were used twice, once without and once with sign inversion of all spectral points (Staykova et al. 2008b). Figure 3 shows as an example the “N + CA/CB” vs. HN plane (diagonal with respect to the nitrogen and carbon axes), where either only the red, positive Cα intensities are considered, or, after inversion, only the blue, negative Cβ intensities. Often, no ppm scale can be given in the indirect dimensions of the projection planes due to combinations of different nuclei types, such as nitrogen and carbon in Fig. 3; thus a point scale is used throughout in the indirect projection dimensions.

### Create intervals; decompose

For each backbone signal in the ^15^N-HSQC plane of the TOCSY, an interval for decomposition was created in the following way. An nmrPipe routine (Delaglio et al. 1995) was used for peak picking of the plane. There were 71 clear signals (significantly stronger than the rest); this is one signal less than expected: 76 residues minus three Pro residues minus the N-terminal residue. We decided to complement with a weaker peak found at 9.37 ppm/107 ppm (which was, together with two artefacts on the line δN = 115 ppm near δHN = 8 ppm, the next largest peak; as expected, the results for this interval turned out to be rather poor). Intervals (along the direct dimension HN) were then defined for each of the 72 signals using the maximum point along the HN axis plus and minus one point (i.e. all intervals were three points broad).

The plane selection (see above) amounts to 19 planes in the first case and 13 planes in the other two cases (note that for HNCACB, three of the six planes are obtained by mere sign inversion). The decompositions provided a shape for each of the nuclei listed in Table 1 for a given experiment combination (nuclei names are explained in a footnote to Table 1), plus a narrow shape for HN. Note that the shape with the TOCSY side chain (aliphatic) carbons is denoted as “Cali” and that with the (aliphatic) side chain hydrogens as “Hali” (all shape names are defined in a footnote to Table 1). Decompositions were run in an automated manner as follows: For all intervals, calculations were started by assuming one component. If a calculation did not converge to an acceptable component according to the following criteria, the number of components was increased by one for a new decomposition until convergence was achieved or a maximum of eight components was reached. Convergence was checked by the following simple procedure: the shape for ^15^N should contain only one maximum (using a cut-off of 10%), and this maximum should coincide, within 0.5 ppm, with the ^15^N shift from the ^15^N-HSQC plane. For the three experiment combinations no convergence was obtained before reaching the maximum of eight components for 2, 2 and 5 intervals, respectively (typically this is due to incomplete separation of components called “mixing”; see Orekhov et al. 2001). This always involves the interval for the weak ^15^N-HSQC peak (see above). In all non-converging cases, the best decomposition with lowest second N peak was selected, yielding second N peaks of 11–21% of the proper one and thus exceeding the cut-off.

### Peak picking in (1D) shapes

Peak picking was applied to all shapes using the same algorithm. Input besides the shapes were the expected maximal number of peaks (e.g. five for the Cali shape, as could occur in a Lys side chain), noise cut-offs (15% for CA and CO, 25% for all others), a minimal difference between two peaks of 0.4 ppm, and shift ranges derived from the BMRB (Ulrich et al. 2008) data base (e.g. 8.7–73.3 ppm for Cali). A maximum is simply a point that is higher than the two neighbors. At this point, the following residue types were identified: Gly based on the Cα shift, and Ala, Ser and Thr based on the Cβ shifts. Tentative assignments were made between peaks indicating the *i − 1* and *i* nuclei by comparing with the Cali shape and/or choosing the stronger peak as belonging to the same residue as the H-N group. After peak picking, the assignment procedure was followed by two mutually independent steps (resulting in a branching in the flow-chart of Fig. 2): (a) identifying compatible residue types for each component and (b) investigating sequential neighborhood of all component pairs.

### Assign residue types to intervals

The identification of compatible residue types was based on a score between the Cali shape of each component and the average expected shifts for a residue type according to the BMRB data base (Ulrich et al. 2008). The score considered (a) if all expected BMRB aliphatic carbon shifts of a given residue type are represented in the shape, (b) if all peaks in a shape can be matched to a BMRB shift, and (c) if the number of observed and expected peaks match. Scores for (a) and (b) are evaluated with the help of Gaussian curves defined by the average shifts and standard deviations given by the BMRB data, as follows: For each expected resonance according to BMRB, a normalized Gaussian curve, centered on the expected chemical shift and width given by the BMRB standard deviation, is defined. Observed peaks receive the value of the curve at the observed shift.

### Sequential connections among intervals

This step includes calculation within a first 2D matrix with the enumeration of intervals on both axes (in the next step, another 2D matrix is defined and used, see below). In this interval–interval matrix, an element at position (*l, k*) describes the probability that interval *l* is succeeded by interval *k*, as follows. Both the HNCACB and the HNCACO provide resonances for nuclei of the residue to which the amide group belongs (residue *i*), and of the preceding residue (*i − 1*), Cα and Cβ, respectively Cα and CO (Fig. 1). Shift coincidence (of a given type of nucleus, e.g. Cβ) between the residue *i* shift of one interval and the residue *i − 1* shift of another indicates a possible sequential arrangement of the two intervals. A function for the quantification of this coincidence provided a value of + 1 up to a first cut-off shift difference of 0.4 ppm, a value of − 1 beyond a second cut-off shift difference of 0.8 ppm, and a smooth transition in between. Shift coincidences of several nuclei were used to increase the probability of the two intervals to be sequential (i.e. to increase the entry (*l, k*)). The resulting 2D matrix was optimized by looking for unique entries on either a row or a column, which resulted in zeroing corresponding rows and columns (except for the unique entry). This matrix, together with the result from the assignment of residue types, was input for the next step.

### Assign intervals to sequence; confirm

The results from the above two steps were combined in a second 2D matrix, where the intervals were enumerated on one axis and the sequence was on the other. Entries indicated possible assignments (missing entries indicated impossible assignments). Thus, an unambiguous overall assignment is obtained when a row or a column contains exactly one entry (except for empty columns below Pro in the sequence). An initial version of this matrix was constructed by using the interval–interval connection data from the previous 2D matrix (see above), but also including possible residue types in these connections (e.g., a possible connection between two intervals, both identified as Ala, was removed if the sequence contains no Ala–Ala fragment). This matrix was then improved by applying some rules (e.g., a single entry on a row assigns an interval unambiguously to a certain residue; as a consequence, this residue cannot be assigned to another interval, i.e. all other values in the corresponding column can be set to zero).

In principle, this step provided the sequential assignment. However, the above rules provide a unique result also when probabilities were rather low, and presenting several alternatives may be more reasonable. The final decision of accepting an assignment or not was therefore made by a separate confirmation program that provides a result “per residue” and is thus more reliable. This program checked all assignment results again comparing the coincidence of chemical shifts that were the basis for sequential connections, i.e. for Cα, Cβ and CO (when available); because CO shifts provide less reliable sequential connections, their contribution to the confirmation was reduced.

### Heavy atom chemical shifts

Assignment of heavy atom chemical shifts was performed separately for backbone nitrogen atoms (taken from the initial ^15^N-HSQC peak list), and for carbons. Within each interval assigned to a sequence residue, all observed Cali resonances (peaks) were optimally matched to expected shifts according to BMRB data (Ulrich et al. 2008).

### ^1^H and final chemical shifts

Because resonances for side chain carbons and hydrogens appear, after the decompositions, in two (TOCSY-) shapes called Cali and Hali, their mutual connections are lost (for example, it is unclear which peaks in the Hali shape belong to hydrogen atoms bound to the Cβ or to the Cγ etc.). In part, this problem can be solved again by matching observed shifts with expected shifts according to BRMB; in fact, this worked rather well for Hα. However, a more complete assignment of the side chains hydrogen atoms is obtained by the previously described procedure DIADECOMP (Fredriksson et al. 2016), which pairs resonances of Hali with those of Cali. Note that this procedure can (and did) complement the above carbon assignment: some missing carbon assignments were added and even a few errors were corrected. Also important is the fact that DIADECOMP does not require any new experiments, i.e. it does not add anything in terms of instrument time (Fredriksson et al. 2016).

## Results

### Decompose, peak picking in shapes

**Fig. 4:**
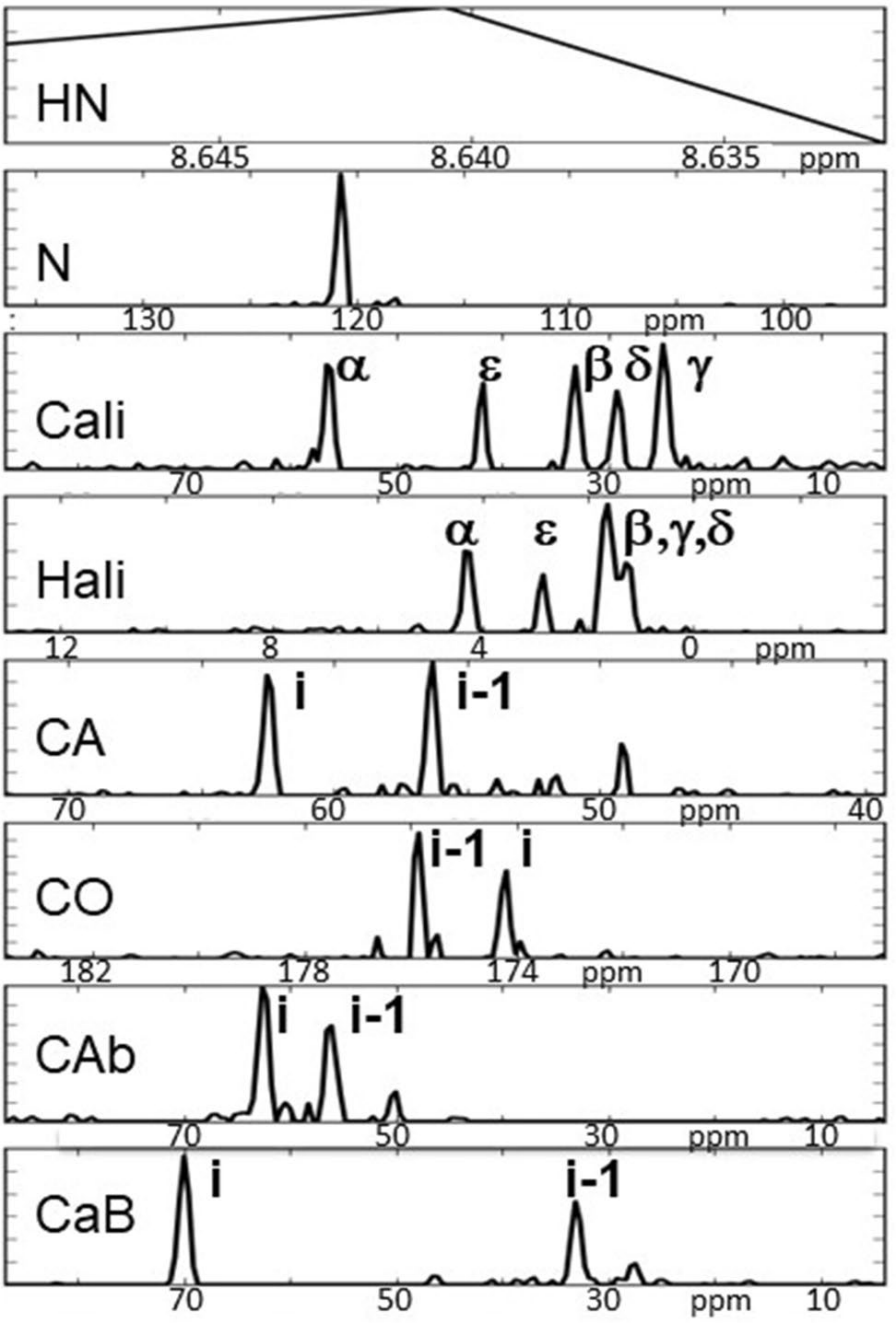
Example component from the “OB” decompositions with the H-N of Thr12 and the side chain of Lys11. The component consists of eight shapes, corresponding to eight different dimensions in conventional experiments. Except for the HN (direct dimension), which consists of three points (see “Methods”), all other dimensions provide full resolution, covering the full spectral width with 192 points (after processing). The Greek letters as well as the position indications (“i”, “i *−* 1”) were obtained after application of all the steps of Fig. 2. These steps also yielded the final assignment to the amide group of Thr12 and the side chain of Lys11. The scale for the axis “CA” (Cα from the HNCACO) differs from the one for the axes “Cali”, “CAb” (Cα from the HNCACB) and “CaB” (Cβ from the HNCACB) because the spectral width of the latter three also includes at least Cβ shifts

Three different combinations, “OB”, “B” and “O”, of the three experiments referred to as TOCSY, HNCACO and HNCACB (see Table 1) were decomposed as described in Methods, yielding in each case 72 components (one for each residue, except Pro residues and the N-terminus). In the following, results are primarily given for the first experiment combination (“OB”), i.e. using a total of 19 planes selected from all three input experiments (Table 1); results for the other two combinations are mostly discussed at the end. Figure 4 illustrates a decomposition result from the combination “OB”; the corresponding interval was later assigned to the H–N of Thr12 and the side chain of Lys11. This decomposition required two components for convergence. (The second component, as well as additional results, for two non-converging decompositions and for an artefact peak of the ^15^N-HSQC plane mentioned earlier, are shown in Fig. S1 for comparison.) Automatic peak picking (see “Methods”), yielded peaks in each shape of each component (Table S5 provides an example). The complete peak list served as input for subsequent steps, i.e. after peak picking no feedback was made to spectral data of projection planes and/or components.

### Assign residue types to each interval

In a first step after peak picking (Fig. 2), the peak list was used to assign compatible residue types to each component. For example, Fig. 4 (and Table S5) clearly describe a Lys side chain (in particular the CεH shifts are unique) of residue *i − 1*. Furthermore, the Cβ shift of almost 70 ppm for the *i* residue identifies it as a Thr. Comparing each of the 72 components with each of the 20 residue types from the BMRB (see “Methods”) resulted in a list with compatible residue types for each interval. Compatible residue types are those with a high score (defined in “Methods”), and the number of compatible residue types for intervals varied between one and six. The distribution of the number of compatible residue types is given in Table 2 for all three experiment combinations.

**Table 2:** Residue type assignment

Number of compatible residue types per interval	Number of occurrences^a^		
	“OB”	“B”	“O”
1^b^	41	42	36
2	14	14	18
3	10	9	8
4	5	5	7
5	2	2	2
6	0	0	1

^a^The sum of the number of occurrences is 72, i.e. the number of intervals

^b^Unique assignments (one compatible residue type) are typically found for Gly, Ala, Ser, Thr but also for example for Lys 11 as shown in Fig. 4 (and Table S5)

### Sequential connections among intervals

A first 2D matrix with interval enumeration on both axes was calculated as described in Methods. This yielded on average for each interval about three possible connections to neighboring intervals. Some intervals were missing any connection, either backward to a preceding interval or forward, but all were in later steps assigned (these later steps also showed that most missing connections were near prolines). Two backward connections as well as two forward connections (not affecting the same intervals) were also corrected in later steps.

### Assign intervals to sequence; confirm

The next step involved again internal work on a second 2D matrix (see “Methods”), this time with the sequence on one axis and intervals on the other; i.e. performing assignments of intervals to residues. In the case where all three experiments were used as input (combination “OB”), each interval was uniquely assigned to one residue. Coupled to these calculations is an analysis step that determines for every sequential connection how reliable it is, i.e. how many coincidences of shifts support the connection. For “OB”, only the first residue and residues preceding Pro (for which no TOCSY information is available) were not confirmed. Thus, the complete interval assignment result was confirmed (which later also turned out to be correct). For the experiment combination “B”, the result is the same, i.e. assignment of intervals to residues in the sequence yields a full assignment. For the combination “O”, 13 intervals were not confirmed, which included eight assignments that were later shown to be incorrect. However, one confirmed assignment turned later out to be also incorrect (Glu16, which remains wrong also in the final results; a likely explanation is missing peaks for Cα in two subsequent residues and also very similar CO shifts).

### Heavy atom chemical shifts

Assignment of chemical shifts was done in different ways for different atom types: HN and N chemical shifts were extracted from the ^15^N-HSQC, and are thus “by definition” correct (but can be listed as missing or differing from a reference if the corresponding interval was not or erroneously assigned as in results for the combination “O”). Cα and Cβ atoms were identified in the corresponding shapes. The same holds for CO atoms, but this data is less reliable due to overlap, and it also cannot be checked using the Cali shapes, where both Cα and Cβ atoms should also occur. Side chain carbon shifts were determined by comparing observed shifts (from peak picking) with expected BMRB data. Side chains hydrogens were assigned as described in the next paragraph.

### ^1^H chemical shifts

**Fig. 5:**
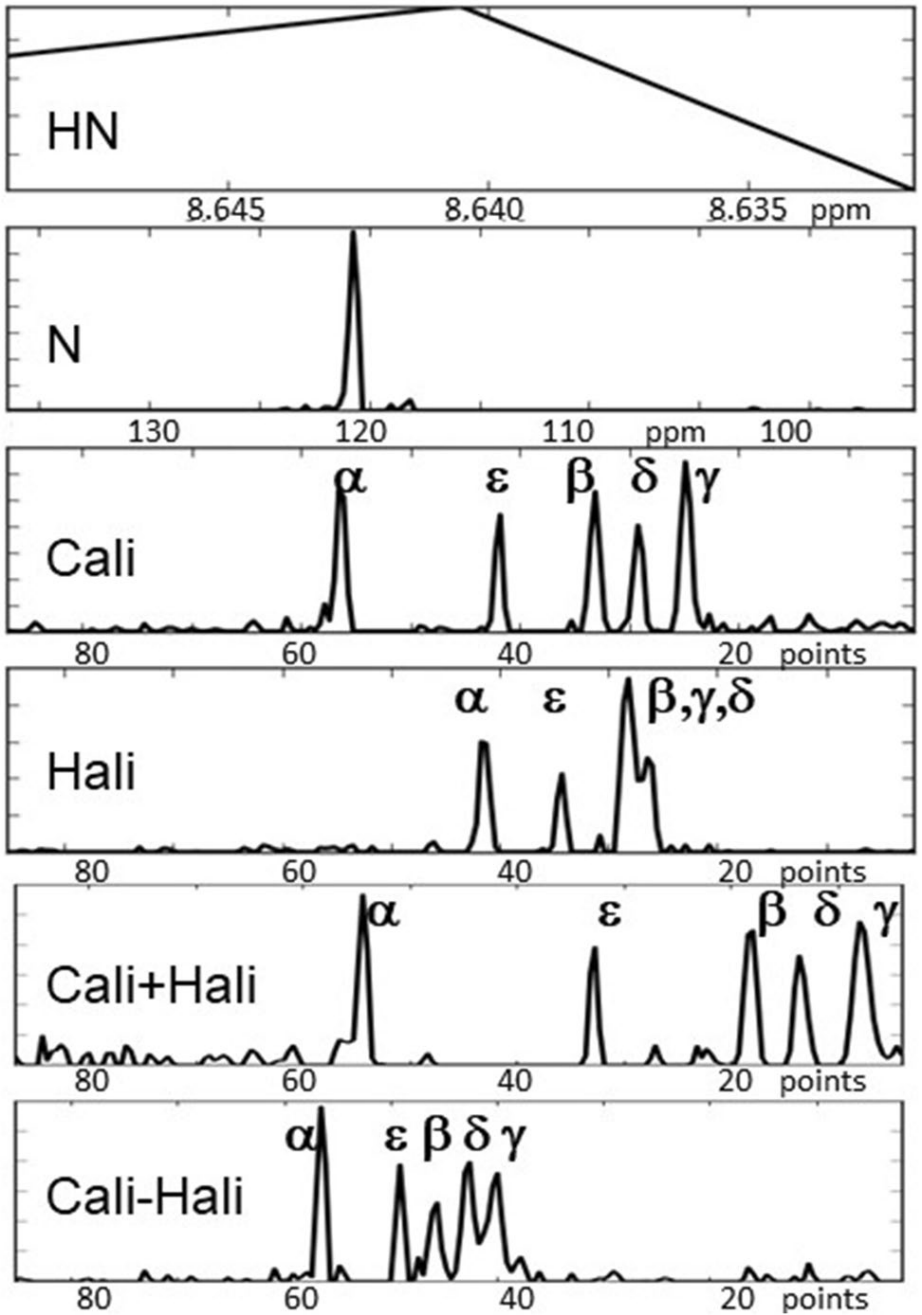
Example of a DIADECOMP (Fredriksson et al. 2016) result for the amide group of Thr12 and the side chain of Lys11 (the first four shapes, including the HN shape, are the same as in Fig. 4). The last two shapes contain DIADECOMP pair resonances of the Cali shape with those of the Hali shape, with the use of the shapes labelled “Cali + Hali” and “Cali − Hali”. The latter were obtained by a decomposition in a rotated 2D frequency space. The numerical matching for this component is given in Table S6. All signals are labelled according to the final assignment. Because of the mixture of carbon and hydrogen shifts in the last two shapes, points rather than ppm are used for the horizontal axes. Points are also used for the Cali and Hali shapes, since sum and differences of resonance shifts in these are calculated to check for matches with the last two shapes

Two independent approaches were used to assign side chain hydrogens; note that at this stage the side chain carbons have already been assigned. A total of 244 hydrogens (or hydrogen groups in the case of methylene or methyl groups) can be maximally assigned; this considers the various length of side chains as well as the non-observability of side chain preceding Pro. In a first approach hydrogen shifts, from the shape Hali (of the TOCSY experiment), were compared with expected values according to BMRB. In a second approach, the earlier published DIADECOMP (Fredriksson et al. 2016) algorithm was used. As an example, the component with N–H of Thr12 and side chains of Lys11, is shown in Fig. 5; the calculation of coordinates in normal and rotated shapes for this residue is given in Table S6. While this second approach yielded in all spectra combinations more assignments, it does not include all assignments obtained by the first approach. Consequently, the results from the two results were merged (no contradiction was observed). These assignment of C–H groups, i.e. of atom pairs, are more reliable than the previous assignments of only carbon atoms. Thus, the joint hydrogen and carbon assignments, mainly achieved with the DIADECOMP procedure, did not only complement, but in a few cases also correct, the previous carbon assignments.

### Final assignment results

Table 3 summarizes the final chemical shift assignments for all three spectra combinations. Here, a nucleus can be either “assigned” or “missed” (first two rows). In addition, the assigned signals were compared to a shift list obtained from the BMRB (entry 6466; Wand et al. 1996; Ulrich et al. 2008): Assignments are either a “match”, “different” (disagreement, meaning that one is wrong), or “additional” (not given in the reference). The last column in the table shows that assignment of hydrogens was less complete than of heavy atoms. Nonetheless, the hydrogen assignment not only yielded no errors, it also improved somewhat the carbon assignments.

**Table 3:** Final chemical shift assignments

Nuclei	HN	N	Cα	Cβ	CO^c^	Sidechain	All C-Hn^d^
Combination “OB”							
Assigned^a^	72	72	74	69	75 (59)	99	186
Missed^a^	0	0	1	0	0 (16)	6	58
Match^b^	72	72	74	68	72 (59)	98	186
Different^b^	0	0	0	1	3 (0)	1	0
Additional^b^	0	0	0	0	0	3	0
Combination “B” (no CO data)							
Assigned^a^	72	72	74	69	–	98	189
Missed^a^	0	0	1	0	–	7	55
Match^b^	72	72	74	68	–	96	189
Different^b^	0	0	0	1	–	2	0
Additional^b^	0	0	0	0	–	3	0
Combination “O”^e^ (Cβ data only via TOCSY)							
Assigned^a^	59	59	62	51	62	81	162
Missed^a^	13	13	13	18	13	24	82
Match^b^	58	58	61	49	61	79	162
Different^b^	1	1	1	2	1	2	0
Additional^b^	0	0	0	0	0	2	0

^a^Numbers in these two rows represent the results of the current assignment. “Assigned” includes all assignments made. Observable nuclei include 72 HN and N, 75 Cα and CO, and 69 Cβ (excluding 6 Gly). In addition, there are in total 105 side chain carbons (with hydrogens) observable (not counting unobservable groups preceding Pro residues)

^b^Numbers in these three rows show a comparison with independent literature data (REF). “Match” and “different” are based on a cut-off of 0.5 ppm (for carbons); “additional” are new assignments not present in the reference data (counted together with “matched” assignments here)

^c^In parentheses are results obtained with stricter demands, where CO connections are required to both neighboring residues

^d^This column reports assignments of C-Hn (n = 1–3) groups via DIADECOMP (REF)

^e^This combination resulted in 13 missing interval assignments (see text) and one erroneous interval assignment (an interval that should have been assigned to Glu16); consequently, an error is reported for all five groups in a Glu

The combination “OB” clearly provides enough data for a complete assignment. Only one α-group (i.e. both Cα and Hα) is missed: Thr22. The initial assignment of carbons, prior to ^1^H consideration, missed seven α-carbons, but all except one were recovered with DIADECOMP; similarly, a single different intermediate α-carbon assignment was corrected by DIADECOMP. Regarding β-carbons, one different assignment remained because DIADECOMP did not assign this group at all. The CO assignments are less reliable, because in contrast to the α- and β-groups they cannot be checked by the type of TOCSY used here. Requiring that CO need to be connected from both sides, removes all three different assignments, however at the expense of 16 missing assignments (Table 3 footnote). Most of the missing assignments in the side chains concern peripheral groups, and so do the three additional assignments (all leucine methyl groups). The results for the “B” combination differ only regarding the side chains. CO assignments are obviously not present. Thus, also this input data is sufficient. However, this is not true for the “O” combination, where 13 intervals could not be assigned reliably to the sequence, and furthermore one interval was erroneously assigned. This yielded at least 13 missed assignments for each nucleus (Table 3), but also at least one difference to the reference. β-carbons show more missing assignments because no direct data (like HNCACB) was in the input, and thus assignments had to be deduced from TOCSY data.

## Discussion

The main objective of this contribution was to present a novel approach in protein NMR consisting of joint analysis of a multitude of spectra recorded for different purposes, but that all are part of a single project. A protein NMR project typically starts with assignments, but the real goals may be structure, dynamics or interaction information. “Projection-decomposition” provides means for rapid recording of data (“projections”), by optimizing the relevant S/N (Malmodin and Billeter 2006): Signals result from the combined analysis of all input planes, allowing to detect resonances that are below the noise level in individual projection planes; this is not possible if peak picking is applied directly to the planes. In addition, it allows joint analysis of all spectra, requesting only that they share the direct dimension; often but not necessarily this is the amide hydrogen dimension. Joint analysis includes not only all projections from a single experiment, but projections from various experiments can be combined, such as triple-resonance like experiments (often in more than three dimensions) with experiments including TOCSY or NOESY sequences (Fredriksson et al. 2012a). Joint decomposition yields two different advantages. (1) Complex spectra, for example spectra with a significant presence of noise, a high dynamic range among the signals and/or a large number of signals, may be supported by spectra lacking these difficulties. (2) Additionally, information from the various spectra are automatically collected in individual “spin systems”: Figs. 1 and 4 illustrate this aspect, where a “spin system” is centered on an amide group (unique resonances for both HN and N in the top two shapes in Fig. 4), and the other shapes present various resonances of neighboring nuclei. (In an earlier publication we presented a figure where the other shapes included triple-resonance type data together with TOCSY and NOESY information: Fig. 1 in Fredriksson et al. 2012a.)

The above features can be used to design a novel, flexible and scalable approach: Selected projections, based on S/N or any other considerations, from several experiments chosen according to the ultimate goal of the project are jointly decomposed to yield 1D shapes that can be analyzed to yield a certain goal/purpose. Flexibility means that the choice of experiments depends on the purpose: backbone assignments will typically rely on triple resonance experiments or derivatives, side chain assignments will usually include a TOCSY-like spectrum, and structural/interaction information is likely to require a NOESY-type experiment. Also, projection selection may avoid repetitions (e.g. only one ^15^N-HSQC plane is needed) or projection planes with low S/N (e.g. due to evolution on all nuclei involved). Scalability is partly demonstrated with the analyses of several combinations. The combination “B” provides complete results, but larger or more disordered proteins may require combination “OB” or other, additional experiments.

The DIADECOMP approach (Fredriksson et al. 2016) solves an inherent problem with the projection-decomposition method by pairing the hydrogens on one axis with their carbons on another axis (here Hali with Cali); these were separated by the decomposition, and without DIADECOMP their pairing is not always trivial. The DIADECOMP results proved essential not only for assigning also side chain hydrogens, but also for complementing, and in a few cases correcting, carbon assignments. It should be noted that DIADECOMP requires no additional instrument time, but rather relies on the same projection planes as the normal decomposition.

Techniques to speed up NMR on proteins focus often on increasing resolution along indirect dimensions beyond what would be possible in a conventional experiment. Obvious considerations regarding S/N when reducing measurement time may often be handled in all techniques in straightforward ways, for example by increasing the number of scans. Non-uniform sampling (NUS) techniques typically rely on randomized sampling of FIDS selected for recording. Much effort has been put in defining various “biases” of the random selection, and the obvious advantage is that the FID selection can be optimized with respect to information content. In contrast to other approaches (Eghbalnia and Markley 2017), the projection-decomposition approach is bound to pre-defined projection planes, or projection angles with respect to the spectral axes of 0°, ± 45° or 90° (but see Malmodin and Billeter 2006). However, data points in the different projection planes are strictly related to each other by linear dependencies in contrast to “randomly” chosen data points. The simultaneous analysis of all projection planes in projection-decomposition presents two more advantages: a significant improvement of S/N over other projection techniques because the overall S/N is relevant and not that of single projection planes (see above and Malmodin and Billeter 2006), and the mutual help that better resolved planes, e.g. from triple-resonance experiments, offer to analyze more complex ones, e.g. from NOESYs.

In the present application, the TOCSY experiment plays a very central role. It is used not only to assign side chains but also to help place components in the protein sequence (including the amide group of the following residue!) and to discriminate between *i* and *i − 1* Cαs or Cβs. Additional spectra mentioned above as a scalability option may for example include an additional TOCSY experiment with a different mixing time. Time saving factors when recording the TOCSY, and in general for 4D experiments, are in our case well over one thousand with respect to conventional spectra with the same resolution and phase cycle (Table S1). As Fig. 3 illustrates, S/N is good in our case, but other proteins may require a larger number of recorded scans, reducing the speed-up factor. Another option could be 5D TOCSY-type experiments, where CO would be added, to make this experiment even more versatile, in particular in view of partly or fully disorder proteins.

Future work will focus on a further generalization of the approach, by compiling sets of both high-dimensional projection experiments and programs that allow recording and analysis of NMR data for any kind of protein characterization. For example, experiments will include in addition to the “proof of concept” presented here also NOESY-type spectra, and the programs will allow peak picking of shapes from all kinds of spectra providing, if required, also signal intensities.

## Conclusions

The main goal here was to introduce a flexible and scalable approach, and to demonstrate in an example, namely the assignment of both backbone and side chain resonances, its applicability. As Table 3 shows, the combination “OB” definitely includes sufficient information to achieve this goal at least for ubiquitin. In fact, the subset in combination “B” is already able to do so, although somewhat less complete intermediate results indicate that the information content is less redundant. Notably, the practically complete result of combination “B” could be achieved with experimental data requiring only about 10 h of measurement (Tables S1 and S2; thus the word overnight in the title of this presentation, which is obviously only true for simple cases like ours). The third combination “O” did not yield as complete results as the other two. On the one hand, this demonstrates that indeed the information content in this combination is lower, as becomes obvious thanks to the use of rather non-sophisticated algorithms. This strongly indicates the (expected) higher value of β-group assignments over CO assignments. However, we cannot exclude that more sophisticated algorithms, for example involving a peak picker that also looks at line widths and symmetry (as many published algorithms do), would not also provide a complete result for the combination “O”. Finally, it should be pointed out that, for the assignment project discussed here, the number of possible errors, or the number of entries in the lines labelled “different” in Table 3, are always very small, even for the “O” combination. The latter combination may be considered not sufficient for a full assignment (too high numbers on the lines labelled “missed”), but this does not lead to a significant number of errors.

## Electronic supplementary material

Below is the link to the electronic supplementary material.

Supplementary material 1 (DOCX 2032 KB)
