# Supplementary material for: Complete protein assignment from sets of spectra recorded overnight
Source: J Biomol NMR. 2019 Feb 15;73(1):59–70. doi: 10.1007/s10858-019-00226-8 (PMC6441399; doi:10.1007/s10858-019-00226-8)
Supplement: Supplementary file 1 — Supplementary material 1 (DOCX 2032 KB) [file 10858_2019_226_MOESM1_ESM.docx]

**Complete protein assignment from sets of spectra recorded overnight**

**Jonas Fredriksson^1^, Wolfgang Bermel^2^, Martin Billeter^1^**

^1^ Department of Chemistry and Molecular Biology, University of Gothenburg, S-40530 Gothenburg, Sweden
^2^ Bruker BioSpin GmbH, D-76287 Rheinstetten, Germany

Corresponding author: Martin Billeter, martin.billeter@chem.gu.se, +46 31 786 3925

**Supplementary Material**

Content

Table S1: Number of FIDS and speed-up factors [Introduction, Methods]

Table S2: List of projection planes and their definition [Methods]

Table S3: Different experiment combinations and corresponding measurement times [Methods]

Table S4: Spectral widths and offsets (ppm) [Methods]

Table S5: Peak picking of interval 10after decomposition of “OB” planes [Results]

Table S6: Addition and subtraction of (point) coordinates in DIADECOMP for Lys11 [Results]

Figure S1: OB decompositions of additional intervals [Results]

a) Second component of the same OB decomposition as in Fig. 4. The proper interval for this spin system is very similar.

b) OB decomposition of the interval that describes Leu15-Glu16 (as shown in the final result). This interval was not converging in the decomposition (see text).

c) OB decomposition of the interval that describes Asp52-Gly53 (as shown in the final result). This interval was not converging in the decomposition (see text), and it corresponds to a weak peak in the ^15^N-HSQC (see text). Note also that glycines have no β-carbons.

d) OB decomposition of an artefact peak of the ^15^N-HSQC (see text). This figure illustrates the noisy shapes from artefacts compared to shapes corresponding to amino acids. This decomposition was run with one component, but using more components yield a similar result.)

**Table S1: Number of FIDS and speed-up factors**

Experiment Dimensions FIDs conventional^#^ Planes^¶^ FIDs in planes^#^ Speed-up^†^

Any 4D spectrum 4 2.1*10^6 13 (all) 1664 1260

TOCSY 4 2.1*10^6 7 (used) 896 2341
(HCCCONH)

HNCACO 4 2.1*10^6 6 (used) 768 2731

HNCACB 3 16384 3 (used) 384 43

^#^ Calculation of number of FIDs is based on recording (or in the conventional case assuming) 64 complex points (other parameters, like relaxation delay or number of scans, cancel in the calculation of a speed-up factor).

^¶^ As described in the text and in Table 1, not all projection planes were used. Note that the unused planes need not be recorded.

^†^ The speed-up factor is obtained by dividing the number of conventionally recorded FIDs by the number of FIDS in used projection planes.

For all experiments: 64 complex points were recorded along all indirect dimensions, and all FIDs were recorded with 16 scans. In a 4D experiment there are 13 planes (3 trivial ones, 6 simple diagonal ones and 4 hyper-diagonal ones); in a 3D there are 4 planes (2 trivial ones and 2 simple diagonal ones). The 4 hyper-diagonal planes of a 4D (those with all three indirect axes involved) are often (like in the present application) not used and thus need not be recorded; in the present application, also the combinations not involving ^15^N were omitted. Finally, the ^15^N-HSQC plane of only one experiment was used, namely the one from the TOCSY (Table 1).

**Table S2: List of projection planes and their definition**

4D TOCSY

The first column enumerates the planes. Sets of planes that are jointly recorded as hyper-complex data sets are between horizontal lines. The four nuclei of the 4D experiment yield the next four columns, with the direct dimension (in parentheses) first. In the projection planes, evolution on selected other nuclei are combined to one indirect axis; the first selected nucleus is marked by “x”. “+x” means that a plane is at 45° with respect to this second (or third) axis and the preceding axis; “-x” indicates an angle of -45°. Planes below the double horizontal line were not used in any of the decompositions.

Plane Nuclei (axes) Comments
 (HN) N Cali Hali

1 (x) x ^15^N-HSQC plane

2 (x) x

3 (x) x

4 (x) x +x These two planes are recorded
5 (x) x -x as one hyper-complex data set

6 (x) x +x These two planes are recorded
7 (x) x -x as one hyper-complex data set

8 (x) x +x Recorded as one hyper-complex data set;
9 (x) x -x both were omitted in the decomposition

10 (x) x +x +x These four planes are recorded
11 (x) x +x -x as one hyper-complex data set;
12 (x) x -x +x all four were omitted in the
13 (x) x -x -x decomposition

4D HNCACO

Same table as above, but with Cali replaced by CA and Hali by CO; the ^15^N-HSQC plane from this experiment is also omitted in all decompositions.

3D HNCACB

Plane Nuclei (axes) Comments
 (HN) N CA/CB

1 (x) x - ^15^N-HSQC plane; omitted in the decomposition

2 (x) - x

3 (x) x +x Recorded as one hyper-complex data set;
4 (x) x -x note that Cα and Cβ have opposite signs

The ^15^N-HSQC plane from this experiment is omitted in all decompositions.

**Table S3: Different experiment combinations and corresponding measurement times**

Combination^¶^ Planes^†^ Time (hours) ^‡^

“OB” 16 16.39

“B” 10 10.27

“O” 13 13.39

^¶^ Experiment combinations are named as follows: Since the TOCSY is part of every combination, no letter is given for it. “O” refers to the HNCACO, and “B” to the HNCACB (thus “OB” refers to the use of planes from all three experiments).

^†^ Planes used for the combination. This refers to a subset of the recorded planes, e.g. only one ^15^N-HSQC was used (see text).

^‡^ These times are extracted from the log files of the measurement (“audita.txt”)

In all experiments, 64 complex points were recorded, each with 16 scans. The number of planes coincides closely with the number of hours used (the average time spent for recording of plane for the combination “OB” is one hour and 1.5 minutes). In the text, recording time for one plane is approximated by one hour (a very slight reduction of the waiting time after each FID would lead to times per plane of less than one hour).

The following planes were NOT used in the assignment according to experiment combinations “OB”, “B” and “O”. TOCSY: indirect axes with shift combinations of Cali and Hali (two planes), and of N, Cali and Hali (four planes); HNCACO: the same (with Cali and Hali replaced by CA and CO) as well as the ^15^N-HSQC plane; HNCACB: the ^15^N-HSQC plane (see also Table S2).

**Table S4: Spectral widths and offsets**

Nucleus spectral width offset

All ^1^H 16.7 4.7

^15^N 41.1 115.9

^13^CO 16.6 175.4

^13^Cα (HNCACO) 33.1 55.8

Other ^13^C 82.8 45.5

In the HNCACO, the ^13^Cα axis includes only Cα shifts. In the HNCACB, the ^13^C axis includes also Cβ and is thus chosen the same as the ^13^C axis for “Cali” in the TOCSY. The values listed above are in ppm.

**Table S5: Peak picking of interval 10**^¶^ **after decomposition of “OB” planes**

Shift Atom Interval i/i-1^†^

8.648 HN 10 i

120.845 N 10 i

24.867 Cali 10 i-1

56.525 Cali 10 i-1

33.106 Cali 10 i-1

41.780 Cali 10 i-1

29.203 Cali 10 i-1

1.628 Hali 10 i-1

4.332 Hali 10 i-1

1.279 Hali 10 i-1

0.849 Hali 10 i-1

62.514 CA 10 i

56.270 CA 10 i-1

174.188 CO 10 i

175.836 CO 10 i-1

62.569 CAb 10 i

56.525 CAb 10 i-1

69.968 CaB 10 i

33.106 CaB 10 i-1

^¶^ The later assignment showed that interval 10 corresponds to the amide group of Thr12 and the side chains of Lys11 (see also Fig. 4).

^†^ “i” indicates atoms of Thr12 (according to the later assignment; “i-1” are atoms of Lys11).

**Table S6: Addition and subtraction of (point) coordinates in DIADECOMP for Lys11**

Cali shape
ppm 56.53 41.78 33.11 29.20 24.87 (α, ε, β, δ, γ)
points^¶^ -26 8 28 37 47

Hali shape
ppm 4.33 2.85 1.63 1.28 (α, ε, βδ, γ)
points^¶^ 4 21 35^‡^ 39

Cali+Hali shape (DIADECOMP)
Points of shape peaks -21 29 63 73 86 (α, ε, β, δ, γ)
Combination^†^ -26+4 8+21 28+35 37+35 47+39

Cali-Hali shape (DIADECOMP)
Points of shape peaks -30 -13 -5 2 8 (α, ε, β, δ, γ)
Combination^†^ -26-4 8-21 28-35 37-35 47-39

^¶^ Conversion ppm to points: points = A*ppm + B with:
A=-mpt/sw;
B=off*mpt/sw+1;
Cali: sw=82.8ppm; off=45.5ppm,
Hali sw=16.7ppm; off=4.7ppm;
mpt: number of points in indirect dimension=192;
sw: spectral width;
off: offset

^‡^ Broad peak, can be two peaks at 34 and 36 points.

^†^ Combinations are within one point (two points for the broad peak in the Hali shape) of the observed resonances in the DIADECOMP shapes.

**Figure S1: OB decompositions of additional intervals**

HN

N

Cali

Hali

CA

CO

CAb

CaB


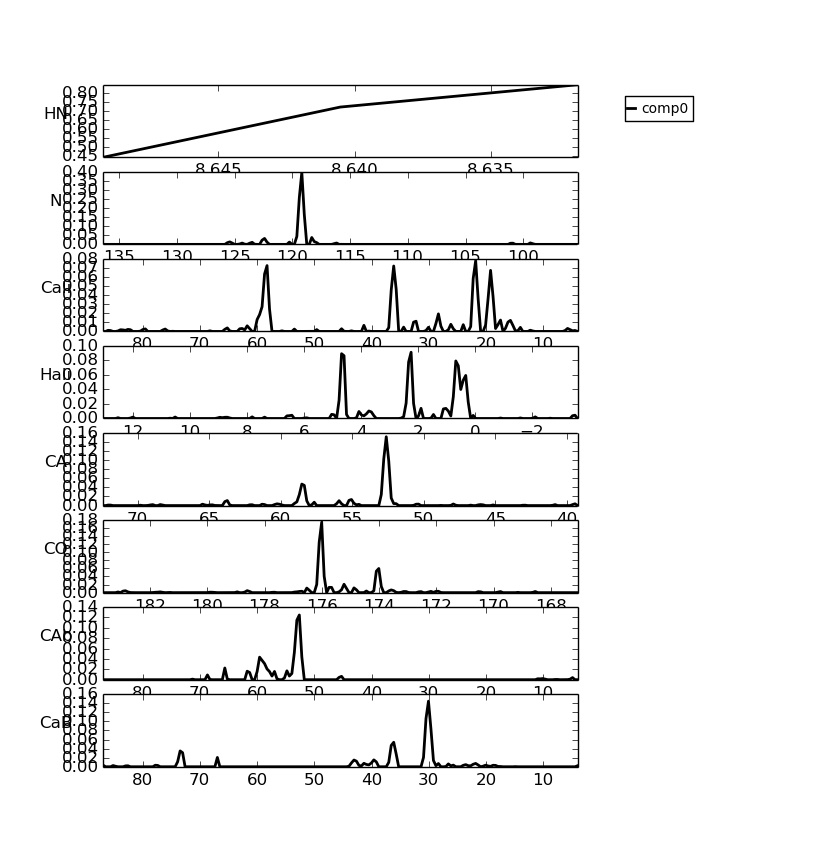


a) Second component of the same OB decomposition as in Fig. 4 (see caption of this figure and Methods for more explanations). The proper interval for this spin system is very similar. (All units on the horizontal axes are ppm.)


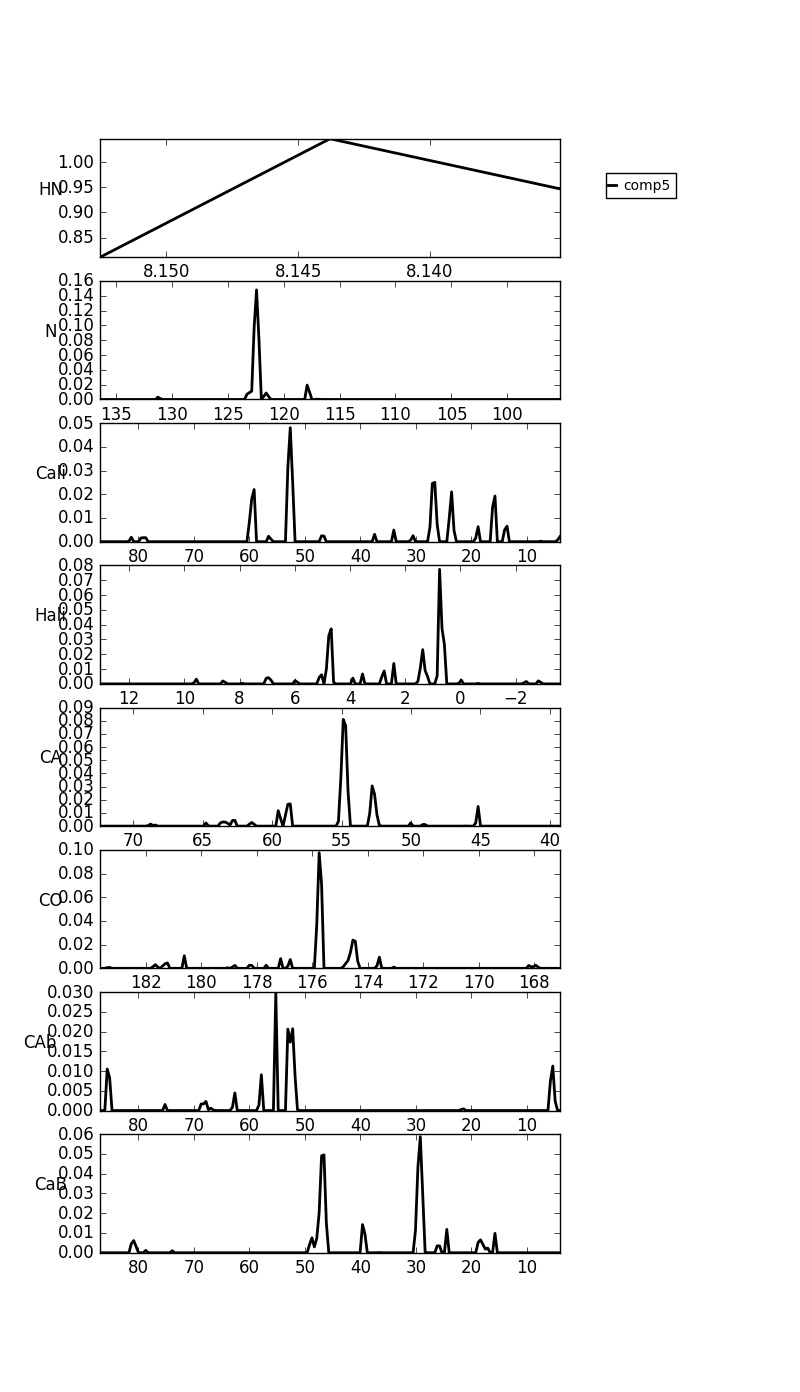


HN

N

Cali

Hali

CA

CO

CAb

CaB

**α γ δ1**

**α γ δ1**

**i i-1**

**i i-1**

**i i-1**

**i-1 i**

b) OB decomposition of the interval that describes Leu15-Glu16 (as shown in the final result). This interval was not converging in the decomposition (see text). (All units on the horizontal axes are ppm.)

.

HN

N

Cali

Hali

CA

CO

CAb

CaB


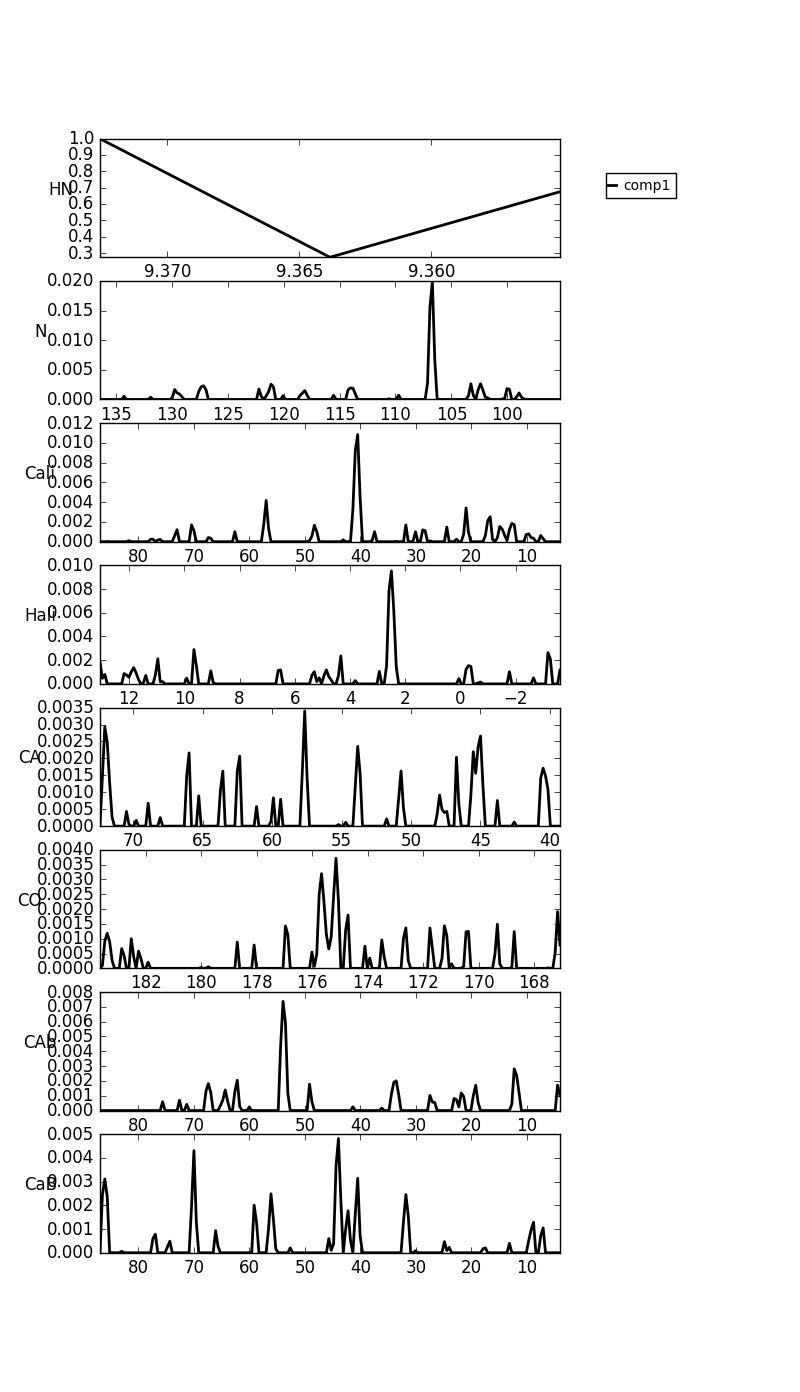


**α β**

**β**

**i-1 i**

**i-1 i**

**i-1**

c) OB decomposition of the interval that describes Asp52-Gly53 (as shown in the final result). This interval was not converging in the decomposition (see text), and it corresponds to a weak peak in the ^15^N-HSQC (see text). Note also that glycines have no β-carbons. (The minimum in the HN shape is a consequence of overlap, and generally of a rather bad decomposition result.) (All units on the horizontal axes are ppm.)

.


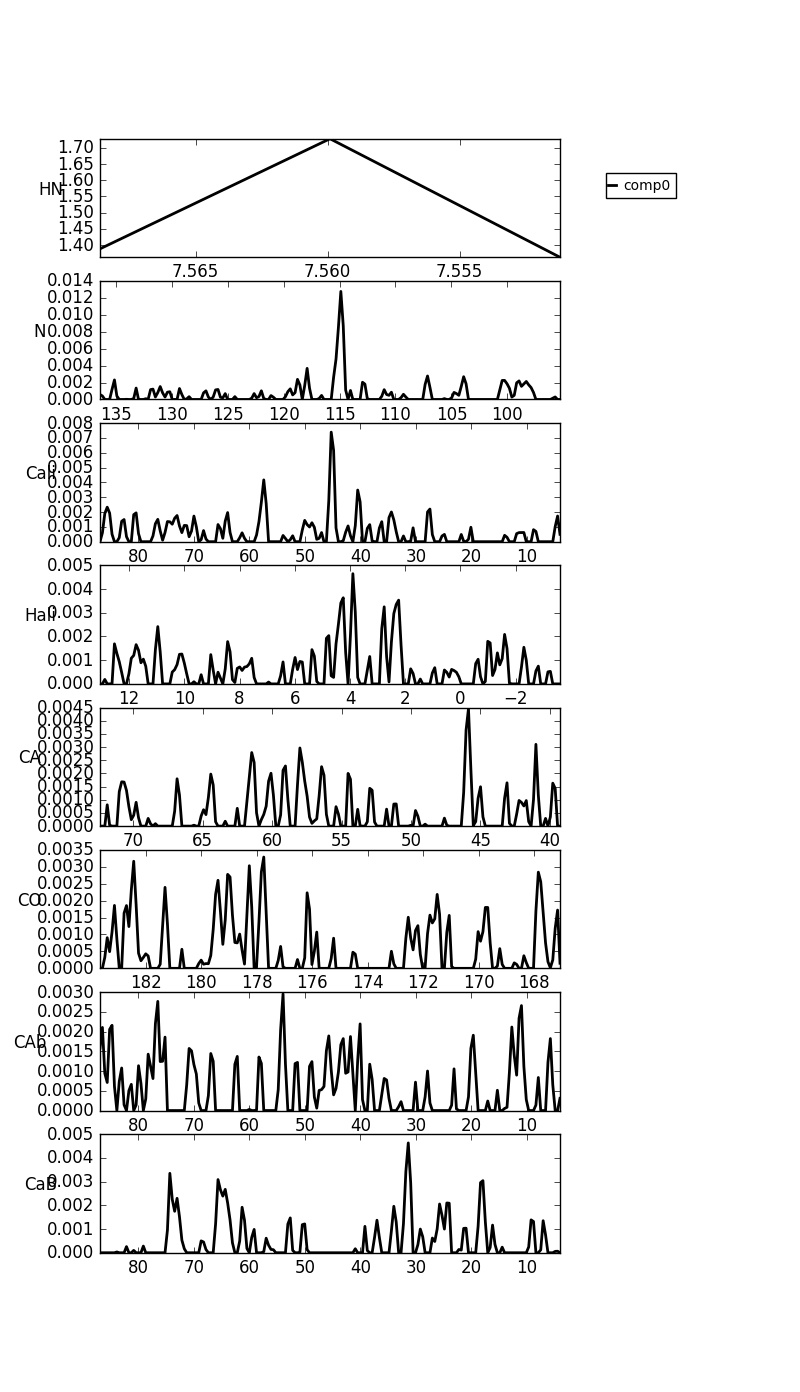


d) OB decomposition of an artefact peak of the ^15^N-HSQC (see text). This figure illustrates the noisy shapes from artefacts compared to shapes corresponding to amino acids. This decomposition was run with one component, but using more components yields a similar result. (All units on the horizontal axes are ppm.)
